# “They might take my baby away:” Black and Latina peoples’ experiences of using cannabis during pregnancy in California while engaged in perinatal care

**DOI:** 10.1038/s41372-023-01781-7

**Published:** 2023-09-20

**Authors:** Rachel Carmen Ceasar, Erin Gould, Jen Laughter, Jordan Granacki, Katherine Kirsch, Edward Chauca, Jasmeen Joy Santos, Lizbeth Becerra, Leticia Cazares, Rima Habre, Shohreh Farzan, Shreya Tamatam, Ryan Mikeala Nguyen, Carrie V. Breton, Theresa M. Bastain

**Affiliations:** 1 Department of Population and Public Health Sciences, Keck School of Medicine, University of Southern California, Los Angeles, CA USA; 2 Department of Sociology, California State University, Fullerton, Fullerton, CA USA; 3 McGill University, Montreal, QC Canada; 4 University of California, Los Angeles, Los Angeles, CA USA

**Keywords:** Medical ethics, Ethics

## Introduction

Low-income and Black, Indigenous, and Persons of Colors (BIPOC) are disproportionately affected by laws that criminalize substance use during pregnancy [1]. Less is known about the unique challenges and stigmas faced by people who use cannabis during pregnancy from their health providers. This qualitative study provides preliminary evidence of the experiences, motivations, and challenges of low-income Black and Latina people who use cannabis during pregnancy and are engaged in perinatal care.

## Methods

We conducted a phenomenological study that aimed to understand the perspectives of a group of people accessing perinatal care in safety net health settings who all experienced a shared phenomenon: using cannabis during pregnancy. As part of the Maternal and Developmental Risks from Environmental and Social Stressors (MADRES) cohort study [2], study staff recruited participants from three recruitment locations that predominantly serve patients with Medi-Cal and were identified as using cannabis during pregnancy via medical record abstraction (*n* = 22). Eligibility criteria included: having given birth in the last 0–3 years, being 21 years or older, and being able to conduct a qualitative interview in either English or Spanish. We conducted 60-min semi-structured interviews (Appendix 1) with Black and Latina people from November 16, 2021, to February 7, 2022, and analyzed data using grounded theory methodology (Appendix 2).

## Results

Of the 22 people identified for the study, one declined stating that they had not used cannabis during pregnancy, 4 were not contacted because they were actively engaged in an existing MADRES study, and 10 were unresponsive, resulting in 7 total participants (Table 1). Further support of the results can be found in Appendix 3: Additional Findings.

**Table 1:** Study participant characteristics (*N* = 7)^a^.

	Mean (SD)/Frequency (%)
Participant characteristics	
Age	27.24 (3.39)
Nativity	
Non-Hispanic	3 (42.86%)
US-Born Hispanic	3 (42.86%)
Foreign-Born Hispanic	1 (14.29%)
Education	
Completed grade 12 (high school)	2 (28.57%)
Some college or technical school	3 (42.86%)
Completed 4 years of college	2 (28.57%)
Income	
Don’t know	2 (28.57%)
Less than $15,000	1 (14.29%)
$15,000 to $29,999	2 (28.57%)
$30,000 to $49,999	2 (28.57%)
Preferred language	
English	7 (100%)
Hispanic ethnicity	
No	3 (42.86%)
Yes	4 (57.14%)
NIH race categories/ethnicity	
Black, non-Hispanic	3 (42.86%)
Hispanic	4 (57.14%)

^a^This pilot qualitative study is part of the broader Maternal and Developmental Risks from Environmental and Social Stressors (MADRES) cohort study that examines critical gaps in understanding the increased risk for maternal and childhood health outcomes among minority and low-income people in urban Los Angeles, California. This small study sample reflects challenges due to recruitment via medical abstraction as well as conflicting cohort study needs.

1. Participants overwhelmingly expressed that they anticipated and/ or felt judgment for their cannabis use, preventing them from having open conversations with their providers.

“… [N]ot a lot of women … come out and say, ‘I use cannabis or CBD oil’ because of that same fear of being looked down upon … They shouldn’t really shame. [Providers] should just monitor how the baby … [and] mom is doing.” (Carmen)

2. Several participants shared that they experienced punitive consequences from their providers because of their cannabis use. For example, one participant who used cannabis in place of Gabapentin for their multiple sclerosis (MS) was reported to the Department of Children and Family Services (DCFS) and required to remain in the hospital an additional day until DCFS approved their release.

“I looked at [the pediatrician] and I was like, ‘Why [did you call DCFS]?’ ‘Oh, well, we found levels of THC.’ I was like, ‘I said that throughout my whole pregnancy [I would be using cannabis for my MS pain], so why are you going to call the police on me and try to get my baby taken away?’ ‘Oh, well, it’s for the benefit of the baby.’” (Linda)

Another participant’s birth experience was impacted on the suspicion that her baby’s father was using cannabis.

“I didn’t smoke [during my first pregnancy], but their father still did. He was there [at the birth] and they felt like he smelled like marijuana, and they made him leave the room [where I was laboring] … They tested me for marijuana and were telling me that if it came back [positive], then that’s what they could possibly do [report me to DCFS] … They told me that they had some of my pee and they were going to test it … I was supposed to be being rushed to get a C-section [when this happened] …[The father] had to wait until the birth was over [before he could join us again].” (Michelle)

Despite not having used cannabis during her first pregnancy, the participant was tested for cannabis and threatened to be reported to DCFS by the labor and delivery team. In addition, her support person was removed from the room and was not permitted to attend the birth of their child.

3. Many participants felt that they were offered few physical and emotional health resources by their providers during their pregnancy. One participant expressed how they trusted cannabis to be a more natural and reliable method than the pharmaceuticals offered by their midwife.

“ ‘Take these pills instead’ … Doesn’t Tylenol and Ibuprofen also damage your liver after taking them daily? … [T]hey would even offer Xanax. Why can’t I just have this [CBD]?” (Isabel)

## Discussion

In this cohort, individuals were often threatened and/or punished by their providers for using cannabis during their pregnancies. We found that people turned to cannabis as an alternative therapy to pharmaceuticals and the medical system, which they felt offered them inadequate care or support during pregnancy. Despite cannabis being legal in California, our preliminary findings demonstrate that the use of cannabis during pregnancy can result in punitive action, including family separation and the involvement of DCFS during and/or after birth.

This study has limitations, particularly the sample size due to recruitment via medical abstraction. Despite the broader MADRES study having obtained informed consent to access information from medical records, some potential participants expressed shock when contacted for a cannabis study, refuting that they had ever used cannabis in the first place. Given that younger, less educated, publicly insured (versus privately insured), and Black (versus White) people are more likely to be asked about substance use during prenatal care visits, and the significant repercussions of selective screening approaches on pregnant people of color, we believe future studies should not recruit people for cannabis studies via medical abstraction alone [3]. Future recruitment efforts would be more ethically and methodologically aligned with our findings by using self-report of cannabis use, despite urine toxicology testing identifying more instances of prenatal cannabis use than self-report.

Despite these limitations, this data suggests the urgency for providers to understand the reporting requirements and policies of both their health system and their local criminal justice system to be more aware of the potential negative consequences overreporting and surveillance can cause for pregnant people and their families, including the possibility of losing patients to care as they may seek treatment elsewhere. With expanding legalization in the U.S., we anticipate that providers and medical systems will be seeing more people using cannabis during pregnancy as prevalence and frequency of prenatal cannabis use have increased in recent years [4, 5]. While it is likely that there are some effects of exposing cannabis to the neonate, research shows there are no clinical benefits to routinely testing mothers and infants for cannabis at the time of birth [6]. As cannabis exposure and its effects on mother and child continue to be studied, it is imperative that providers and medical systems be a non-judgmental, non-biased source of education and information on cannabis use during pregnancy for patients and preserve continuation of care despite substance use.

### Supplementary information

Appendix 1, Interview guide

Appendix 2, Codebook

Appendix 3, Additional findings

## Data Availability

The data generated during this study are available from the corresponding author on reasonable request.
