# Supplementary material for: “They might take my baby away:” Black and Latina peoples’ experiences of using cannabis during pregnancy in California while engaged in perinatal care
Source: J Perinatol. 2023 Sep 20;43(12):1497–9. doi: 10.1038/s41372-023-01781-7 (PMC10716035; doi:10.1038/s41372-023-01781-7)
Supplement: Supplementary file 1 — Appendix 1, Interview guide [file 41372_2023_1781_MOESM1_ESM.docx]

**SETUP**

- The listening session will be about 60 minutes.
- Consent: we will be recording audio and/or video for documentation purposes via Zoom.
- We may interrupt you to be mindful of your time and to get to all of our questions.
- There are no right / wrong answers; we just want to get your perspective. Assume I’m naïve!

**PURPOSE**

- Learn about your prenatal experience.
- Better understand cannabis use and use patterns during pregnancy and early parenthood.
- Identify opportunities to improve maternal health + knowledge about women’s cannabis use during pregnancy.

--------------------------------------------------------- **RECORD** ----------------------------------------------------

**0:00**  **(10 min) BACKGROUND: Understanding origin story**

- **Icebreaker**: name + most proud moment in the last year as a parent during the pandemic
- Tell me about the birth of your baby.
  - How did you prepare for the birth? (probe: family dynamics, household, neighborhood, support system, prenatal care access / costs, barriers to care)

**0:10 (20 min)** **UNDERSTANDING PRENATAL CANNBIS USE: Perceptions + knowledge**

- Tell me about the first time you used cannabis.
- What were your motivations for using cannabis?
- How did those motivations change during pregnancy + parenthood? (probe: nausea, vomiting, and depression related to pregnancy; cannabis beliefs and norms)
  - How did your use change / differ than before? (probe: use patterns, frequency)
  - What products do you prefer to use / not use during pregnancy + parenthood? (probe: CBD/THC, smoke, oils, edibles, grow own)
  - What are some of the pros / cons to using that kind of cannabis?
  - How easy / hard is it to get cannabis where you are? (probe: online, corner shop, trades)

**0:30 (20 min) UNDERSTANDING SOCIAL ENVIRONMENT ON CANNABIS USE: Influencers + social factors**

- Who did you prefer to use cannabis with + when? (probe: partner, coworkers, to wake up)
- How did you learn about different cannabis products for pregnancy? (probe: familial/peer influences, social media, influencers, corner shop)
  - How did you figure out which information is helpful and which is not?
  - Have you talked to anyone else about your cannabis use? (probe: nurse, social worker, health care provider; family / partner / friends, OB-GYN)
  - What other substances did you find helpful during pregnancy + parenthood?

**0:50 (10 min) EXPECTATIONS & WRAP UP**

- What do you wish you could tell your doctor about women who use cannabis during pregnancy?
- What do you wish you could tell your partner / family / friends about cannabis use during pregnancy?
- Final thoughts + questions for us?

Additional questions:

**Developed by [Author’s name] using the following resources:**

Jarlenski M, Tarr JA, Holland CL, Farrell D, Chang JC. Pregnant Women's Access to Information About Perinatal Marijuana Use: A Qualitative Study. Womens Health Issues. 2016 Jul-Aug;26(4):452-9. doi: 10.1016/j.whi.2016.03.010. Epub 2016 May 4. PMID: 27131908; PMCID: PMC4958505.

Latuskie KA, Andrews NCZ, Motz M, Leibson T, Austin Z, Ito S, Pepler DJ. Reasons for substance use continuation and discontinuation during pregnancy: A qualitative study. Women Birth. 2019 Feb;32(1):e57-e64. doi: 10.1016/j.wombi.2018.04.001. Epub 2018 Apr 16. PMID: 29673617.
