# Supplementary material for: “They might take my baby away:” Black and Latina peoples’ experiences of using cannabis during pregnancy in California while engaged in perinatal care
Source: J Perinatol. 2023 Sep 20;43(12):1497–9. doi: 10.1038/s41372-023-01781-7 (PMC10716035; doi:10.1038/s41372-023-01781-7)
Supplement: Supplementary file 2 — Appendix 2, Codebook [file 41372_2023_1781_MOESM2_ESM.docx]

**People who use cannabis during pregnancy study (2021-2022)**

**Codebook (16 codes)**

Format:

[[**CODE NAME**]]: Definition

*Examples: data-driven instances / experiences from transcripts*

**RESEARCH AIM:** Evaluate themes surrounding people’s perceptions and knowledge of cannabis use during pregnancy, encompassing domains in: social environment influences (e.g., family and peers), norms and intentions, personal and social barriers, background influences, beliefs and motivations

**Global codes (4)**

**Race:** discussion of race or ethnicity in cannabis x pregnancy

*Example: health systems targeting people of color*

**Class:** discussion of socioeconomic status or related class structures in cannabis x pregnancy

*Example: receiving low-income services and how impact cannabis use consequences; being poor and being harassed for use*

**Postpartum:** discussion of postpartum period and cannabis use decision making

**Good quote:** key quote that provides insights into research questions

**Learning about and accessing cannabis during pregnancy (3)**

**Gaining knowledge about cannabis:** receiving advice and info about cannabis use for pregnancy

*Example: from dispensary where I work; friends; own research, doula influencers on IG, books, learned pre-pregnancy about usefulness for nausea symptoms, social media*

**Describing facilitators + barriers to getting cannabis:** pros/cons to getting cannabis for use during pregnancy

*Examples: access with legalization, can have harmful effects, expensive, buying from street vs dispensary*

**Describing desired relationship with cannabis:** describing how perceive cannabis use during pregnancy

*Examples: partying vibe, community mindset; keeping baby healthy, moderation ok for symptom relief; sharing what’s acceptable; approaching sativa as related to health; overusing self-reflection, abusing vs medicating/healing*

**Patterns of use during pregnancy (3)**

**Altering use patterns**: changing cannabis use patterns, administration, and/or frequency across pregnancy

*Example: topicals instead of smoking, smoking instead of edibles; topicals v smoking; helpful non-inhaled methods*

**Avoiding use:** decision making around reduction or stopping use of cannabis across pregnancy

*Examples: stopping to keep family happy; creating smoking routine to cut back, avoiding during nursing*

**Comparing cannabis to other substances**: comparing cannabis use to tobacco, other substances, during pregnancy

*Examples: noting cannabis better to use than tobacco, vaping during pregnancy*

**Motivations for cannabis use during pregnancy (3)**

**Describing motivations for physical relief:** explaining why use / not use during pregnancy for physical relief

*Examples: reducing pain for MS, sleep, morning sickness, part of everyday routine; being disabled*

**Describing motivations for emotional relief:** explaining why use / not use during pregnancy for mental health

*Examples: anxiety, coping, stress relief, feeling unstable with life; Reducing hardships at work; b/c poor mental health access; prevent postpartum depression*

**Using cannabis instead of...:** comparing why using cannabis instead of pharmaceuticals / what provider recommended

*Examples: instead of MS drugs, comparing to pharma drugs, mixing cannabis with Motrin, use like Tylenol; run bath; iron pills; recommended Xanax but thought too strong; pills to stimulate appetite; nausea pills that made her throw up so use cannabis instead*

**Challenges to cannabis use during pregnancy (3)**

**Describing relationship with healthcare surrounding cannabis use:** explaining provider / health system challenges with use of cannabis during pregnancy

*Examples: lack of open dialogue, not receiving info; scare tactics; being honest about use, feeling safe to disclose; wanting provider to put deadline in place to stop use;* diff protocols around hospital unclear, perceiving healthcare as not safe space

**Experiencing consequences of cannabis use:** describing consequences of cannabis use during pregnancy

*Examples: child services, losing baby to DFCS after birth, getting reported to social workers*

**Experiencing judgment for use**: feeling stigmatized, discriminated against for use of cannabis during pregnancy

*Examples: receiving negative comments at work, looked down upon by partners/family*
