# Supplementary material for: “They might take my baby away:” Black and Latina peoples’ experiences of using cannabis during pregnancy in California while engaged in perinatal care
Source: J Perinatol. 2023 Sep 20;43(12):1497–9. doi: 10.1038/s41372-023-01781-7 (PMC10716035; doi:10.1038/s41372-023-01781-7)
Supplement: Supplementary file 3 — Appendix 3, Additional findings [file 41372_2023_1781_MOESM3_ESM.docx]

Appendix 3, Summary of key findings and recommendations.

| **Results section** | **Key quotes** | **Recommendations** |
| --- | --- | --- |
| 1. Feeling judged and threatened with punitive action by providers for using cannabis during pregnancy | “…[N]ot a lot of women...come out and say, ‘I use cannabis or CBD oil’ because of that same fear of being looked down upon...They shouldn’t really shame. [Providers] should just monitor how the baby...[and] mom is doing...[S]o I didn’t really talk to [my providers] about [cannabis use]...They do questionnaires [patient in-take forms], so they do have it [cannabis listed] on the papers [medical records]. I would just check out ‘No’ to avoid the conversation of them telling me not to use it...I know that it helps me, I’m still going to use it. I felt like there was no point on letting them know.” (Carmen)  “I know if I would have still been smoking [cannabis throughout my pregnancy], hospitals aren't going to play with you if they find marijuana in the system of your child.” (Jamie)  “Here I am getting low-income services and [providers are] quick to threaten me, like, ‘We don’t know why you’re using cannabis, but it’s coming out in your urine, and a social worker could go to your house or they can go to the hospital.’ That was scary because I was like, ‘Wait, there’s a possibility that they might take my baby away?’...It’s really interesting how marginalized communities or poor people get this kind of harassment.” (Marcela) | There is a need for providers to understand the reporting requirements and policies of both their health system and their local criminal justice system to be more aware of the potential negative consequences their overreporting and surveillance can cause for people who are pregnant and their families. |
| 2. Subjected to family separation and additional surveillance by providers for using cannabis during pregnancy | “I looked at [the pediatrician] and I was like, ‘Why [did you call DCFS]?’ ‘Oh, well, we found levels of THC.’ I was like, ‘I said that throughout my whole pregnancy [I would be using cannabis for my multiple sclerosis pain], so why are you going to call the police on me and try to get my baby taken away?’ ‘Oh, well, it's for the benefit of the baby.’...They couldn't do anything because it's a known fact that people are using [cannabis] to get better and it's starting to be legalized and you're still trying to take my baby away for your old mentality ways.” (Linda)  “I didn’t smoke [during my first pregnancy], but their father still did. He was there [at the birth] and they felt like he smelled like marijuana, and they made him leave the room [where I was laboring]...They tested me for marijuana and were telling me that if it came back [positive], then that’s what they could possibly do [report me to DCFS]...They told me that they had some of my pee and they were going to test it...I was supposed to be being rushed to get a C-section [when this happened]…[The father] had to wait until the birth was over [before he could join us again].” (Michelle) | There is a need for future provider and policy interventions that aim to protect patients, particularly Black and Latina patients, from discrimination and criminalization during pregnancy and preserve continuation of care despite substance use. |
| 3. Approaching cannabis as an alternative therapy during pregnancy | “ ‘Take these pills instead’...Doesn’t Tylenol and Ibuprofen also damage your liver after taking them daily?...[T]hey would even offer Xanax [a fast-acting tranquilizer taken by mouth to manage anxiety disorders] ...strong drugs. Why can't I just have this [CBD]?” (Isabel)  “Once I got pregnant...They just said that I couldn't take those [medications for physical and mental health]. Okay, cold turkey it is...I was going to take care of it myself... I hate having to talk to new people about my [mental health] issues [each time I schedule a therapy session] ...They’re student doctors, so every six months [the therapist] changes. I tried going to [a local] mental health [clinic] but it was during the pandemic, [so] they didn't offer any telehealth. It was just a phone call—they'd be like ‘Oh, how are you?’ Five minutes later, ‘Okay, I'll talk to you next time’...[Y]ou're not helpful at all either. I just rather keep it [my mental health issues] to myself then. I've been able to manage [my mental health by using cannabis] I'm not going all crazy like I used to be...That's what's been the most helpful. So I've just kept going with it [during pregnancy].” (Linda) | It is imperative that providers and medical systems be an open, supportive, non-judgmental, and non-biased source of education and information on cannabis use during pregnancy for patients. |
